# CircSAMD4A aggravates H/R‐induced cardiomyocyte apoptosis and inflammatory response by sponging miR‐138‐5p

**DOI:** 10.1111/jcmm.16093

**Published:** 2020-11-21

**Authors:** Xiaorong Hu, Ruisong Ma, Jianlei Cao, Xianjin Du, Xinyong Cai, Yongzhen Fan

**Affiliations:** ^1^ Department of Cardiology Zhongnan Hospital of Wuhan University Wuhan China; ^2^ Department of Cardiology Tongren Hospital of Wuhan University Wuhan China; ^3^ Department of Emergency Renmin Hospital of Wuhan University Wuhan China; ^4^ Department of Cardiology Jiangxi Provincial People's Hospital Affiliated to Nanchang University Nanchang China

**Keywords:** acute myocardial infarction, apoptosis, CircSAMD4A, H/R, inflammatory response, miR‐138‐5p

## Abstract

Hypoxia/reoxygenation (H/R)‐induced myocardial cell injury is the main cause of acute myocardial infarction (AMI). Many proofs show that circular RNA plays an important role in the development of AMI. The purpose of this study was to investigate the role of circSAMD4A in H/R‐induced myocardial injury. The levels of circular SAMD4A (circSAMD4A) were detected in the heart tissues of AMI mice and H/R‐induced H9C2 cells, and the circSAMD4A was suppressed in AMI mice and H/R‐induced H9C2 cells to investigate its’ function in AMI. The levels of circSAMD4A and miR‐138‐5p were detected by real‐time quantitative PCR, and MTT assay was used to detect cell viability. TUNEL analysis and Annexin V‐FITC were used to determine apoptosis. The expression of Bcl‐2 and Bax proteins was detected by Western blot. IL‐1β, TNF‐α and IL‐6 were detected by ELISA kits. The study found that the levels of circSAMD4A were up‐regulated after H/R induction and inhibition of circSAMD4A expression would reduce the H/R‐induced apoptosis and inflammation. MiR‐138‐5p was down‐regulated in H/R‐induced H9C2 cells. circSAMD4A was a targeted regulator of miR‐138‐5p. CircSAMD4A inhibited the expression of miR‐138‐5p to promote H/R‐induced myocardial cell injury in vitro and vivo. In conclusion, CircSAMD4A can sponge miR‐138‐5p to promote H/R‐induced apoptosis and inflammatory response.

## 1 INTRODUCTION

Acute myocardial infarction (AMI) is myocardial necrosis caused by acute and persistent ischaemia, and the development of AMI is related to genetic and environmental factors.^1^ As one of the common cardiovascular diseases, AMI has the characteristics of high morbidity and high mortality.^2^ At present, reperfusion is a common treatment for AMI, but the method will cause damage to myocardial cells.^3^ The hypoxia/reoxygenation (H/R) injury and apoptosis of myocardial cells will bring irreversible damage to the heart, which is an important reason for the occurrence of AMI.^4^ In addition, myocardial cells with H/R injury will go through the processes like inflammation,^5^ and inflammatory response mediates reaction between various injury factors and myocardial injury during the development of AMI.^6^, ^7^ More and more evidences showed the importance of apoptosis and inflammatory response in the pathogenesis of AMI.^8^ Therefore, finding relevant markers that regulates apoptosis and inflammatory response under myocardial H/R injury has positive significance for the treatment of AMI.

Non‐coding RNA is a regulator of gene expression, including microRNA, long non‐coding RNA and circular RNA.^9^ Previous studies found that long non‐coding RNA ROR could promote H/R‐induced cardiomyocytes apoptosis, which was associated with its increased phosphorylation of p38 and ERK1/2 and its combination with miR138 to up‐regulate the expression of Mst1.^10^, ^11^ Recently, circular RNA has become increasingly prominent in the study of myocardial infarction. Circular RNA can interact with specific microRNA to prevent the translation of microRNA, and this process is involved in biological processes such as cell proliferation, apoptosis, autophagy and pathological processes such as cardiovascular diseases, neurological diseases, cancer and other diseases.^3^ Especially in cardiovascular diseases, the circular RNA Cdr1as inhibits miR‐7a through PARP and SP1 and induces apoptosis of cardiomyocytes when myocardial infarction damage is aggravated.^12^ Circular RNA MACF1 can regulate the miR‐500b‐5p/EMP1 pathway to inhibit the occurrence of AMI.^13^ However, the role and mechanism of circSAMD4A in H/R‐induced myocardial injury is not clear.

In this work, we first reported that circSAMD4A was raised in vivo and in vitro studies in AMI. Depressing the expression of circSAMD4A inhibited the apoptosis of cardiomyocytes and the expression of inflammatory cytokines, and we proved that circSAMD4A restrained the expression of miR‐138‐5p to promote cardiomyocytes apoptosis and inflammatory development in H/R‐stimulated H9C2 cells and AMI mice. This finding may provide new ideas for the treatment of patients with AMI.

## 2 MATERIALS AND METHOD

### 2.1 Mice

The 30 male C57BL/6J mice used in this study were purchased from the Model Animal Research Institute of Nanchang University. They were completely randomly divided into a sham group (n = 6), AMI group (n = 6), AMI + si‐NC group (n = 6), AMI + si‐Circ group (n = 6) and AMI + si‐Circ + anti‐miR (n = 6). All animal studies meet the standards in the "Guidelines for Laboratory Animal Care and Use" published by the National Institutes of Health and are approved by the Animal Care and Use Committee.

In order to construct AMI mice model, firstly mice were anaesthetized by intraperitoneal injection of pentobarbital sodium (50 mg/kg, P3761, Sigma‐Aldrich), then, mice were placed on the operating table, and connecting the ventilator after intubation and cut the left sternum of mice were cut to fully expose the heart. The left anterior descending coronary artery was ligated under a microscope to observe the discoloration of the myocardium and identify ischaemia. Adenovirus carrying shRNA against circSAMD4A was injected into mice by myocardial injection to construct a model of AMI + si‐Circ group, and the corresponding control is AMI + si‐NC group. Mice without ligation of the anterior descending branch of the left coronary artery were the sham group in this study. At 2 days after surgery, all mice were killed and hearts were collected and fixed with paraformaldehyde for subsequent studies.

### 2.2 Cell culture and transfection

Rat cardiomyocyte H9C2 cells were cultured in DMEM (Gibco) supplemented with 10% FBS (Gibco). H9C2 cells in the control group were cultured at 37°C and 5% CO_2_. In order to construct a cell model of myocardial H/R injury, H9C2 cells were cultured in a hypoxic incubator (37°C, 95% N_2_ and 5% CO_2_) for 2 hours, and then, the cells were cultured with fresh medium and reoxidated for 4 hours at mixed gas environment (75% N_2_, 20% O_2_ and 5% CO_2_).

The circSAMD4A sequence was cloned into pcDNA3.1, and then, Lipofection 3000 reagent was used for cell transfection. The Liposome 3000 reagent was used to transfect the miR‐138‐5p mimic and its negative control. The transfection time of cells without H/R treatment was 48 hours, but for the cells requiring H/R treatment, cells are treated with H/R 48 hours after transfection.

### 2.3 Real‐time quantitative PCR

Firstly, total RNA was extracted from cardiomyocytes or heart tissues with TRIzol reagent (Invitrogen), and then reversely transcribing total RNA into complementary DNA using a reverse transcription kit (TaKaRa). The expression of circSAMD4A (forward: 5′‐ACTGGCAGGACAAAAGCATG‐3′, reverse: 5′‐CAGGATTTTGGGCAGCAGTT‐3′) and miR‐138‐5p (forward: 5′‐AGCTGGTGTTGTGAATCAGGCCG‐3′, reverse: 5′‐AACGCTTCACGAATTTGCGT‐3′) was measured using the StepOnePlus™ Real‐Time PCR system (application biological system). Relative levels were measured using the 2^−ΔΔCt^ method. The circular RNA levels were normalized to GAPDH (forward: 5′‐CAGGAGGCATTGCTGATGAT‐3′, reverse: 5′‐GAAGGCTGGGGCTCATTT‐3′) or U6 (forward: 5′‐CTCGCTTCGGCAGCACA‐3′, reverse: 5′‐AACGCTTCACGAATTTGCGT‐3′) shRNA.

### 2.4 Western blot

Proteins were extracted from cardiomyocytes or heart tissues with RIPA lysis buffer (Beyotime Biotechnology). Protein samples (60 μg) were separated by SDS‐PAGE electrophoresis and transferred to PVDF membranes (Millipore). After blocking, the protein on the membrane was incubated with primary antibody Bcl‐2 antibody (Cell Signaling Technology) and Bax antibody (Cell Signaling Technology) at 4°C overnight, and then incubated with HRP‐conjugated secondary antibody the next day. Then FluorChemE imager (Alpha) was used for visualization, and the expression level of specific protein was normalized to GAPDH level.

### 2.5 MTT

The cardiomyocytes were seeded in 96‐well plates and incubated overnight at 37°C. Then, the cells were treated according to the instructions, 20 μL of MTT solution (5 mg/mL, Sigma‐Aldrich) was added, incubated at 37°C for 4 hours. Then adding 100 μL of DMSO (Sigma‐Aldrich) to each well. After the operation is completed, the absorbance was measured with a micro plate reader (BioTek Instrument) at 490 nm to determine the viability of the cells.

### 2.6 ELISA analysis

The detection of IL‐1β, TNF‐α and IL‐6 in cells was operated by ELISA kits (R&D Systems). The supernatants of cell culture medium were collected and then centrifuged (2458 *g*, 15 minutes). Following the instructions, each experiment was done for 3 times.

### 2.7 Detection of apoptosis level

To investigate apoptosis, we applied the Annexin V‐FITC apoptosis detection kit (Sigma‐Aldrich, St. Louis, MO). According to the instruction, the cells were collected, washed twice with cold PBS, suspended in 1× binding buffer and stained with 5 µL Annexin V‐FITC for 15 minutes, then cells were stained with 5 µL PA for 10 minutes under dark conditions at room temperature. Finally, the cells were detected by FACSCanto II flow cytometry (BD Biosciences).

### 2.8 TUNEL assay

Apoptosis analysis of cardiomyocytes in heart slices was achieved using terminal deoxynucleotide transferase‐mediated dUTP nick end labelling (TUNEL) staining method by in situ cell death detection kit (RocheFrance). For the heart slices fixed with paraformaldehyde, firstly permeating them with 0.1% Triton X‐100 for 2 minutes, then adding the TUNEL reaction mixture and incubating for 1 hour to stain the apoptotic cells. All nuclei were stained with DAPI. The three random fields were observed in each sample under the microscope, and the percentage of the number of positive apoptotic cells in the total number of nuclei were calculated.

### 2.9 Dual‐luciferase reporter detection

The RNAInter online tool (http://www.rna‐society.org/raid/search.html) was used to predict the binding site of the circSAMD4A and miR‐138‐5p. The circSAMD4A fragment was cloned into pmirGLO vector (Promega) to generate a reporter vector wild‐type circSAMD4A (WT‐circSAMD4A). In order to generate the mutant circSAMD4A reporter vector (MUT‐circSAMD4A), a site mutation is performed. Then, the vectors WT‐circSAMD4A or MUT‐circSAMD4A were co‐transfected with miR‐138‐5p mimics and NC for 48 hours. The cells were lysed, the luciferase activity was detected using the Dual‐Luciferase Reporter Assay Kit (Promega), and the firefly luciferase activity was normalized to Renilla luciferase activity.

### 2.10 Data analysis

All data were analysed with GraphPad Prism 7.04 software (GraphPad Software, Inc), and all experiments were repeated 3 times independently. The experimental results are expressed in mean ± SD, and *P* < .05 indicates a significant difference between the data.

## 3 RESULTS

### 3.1 Increased levels of circSAMD4A in cardiomyocytes in AMI

**Figure 1:**
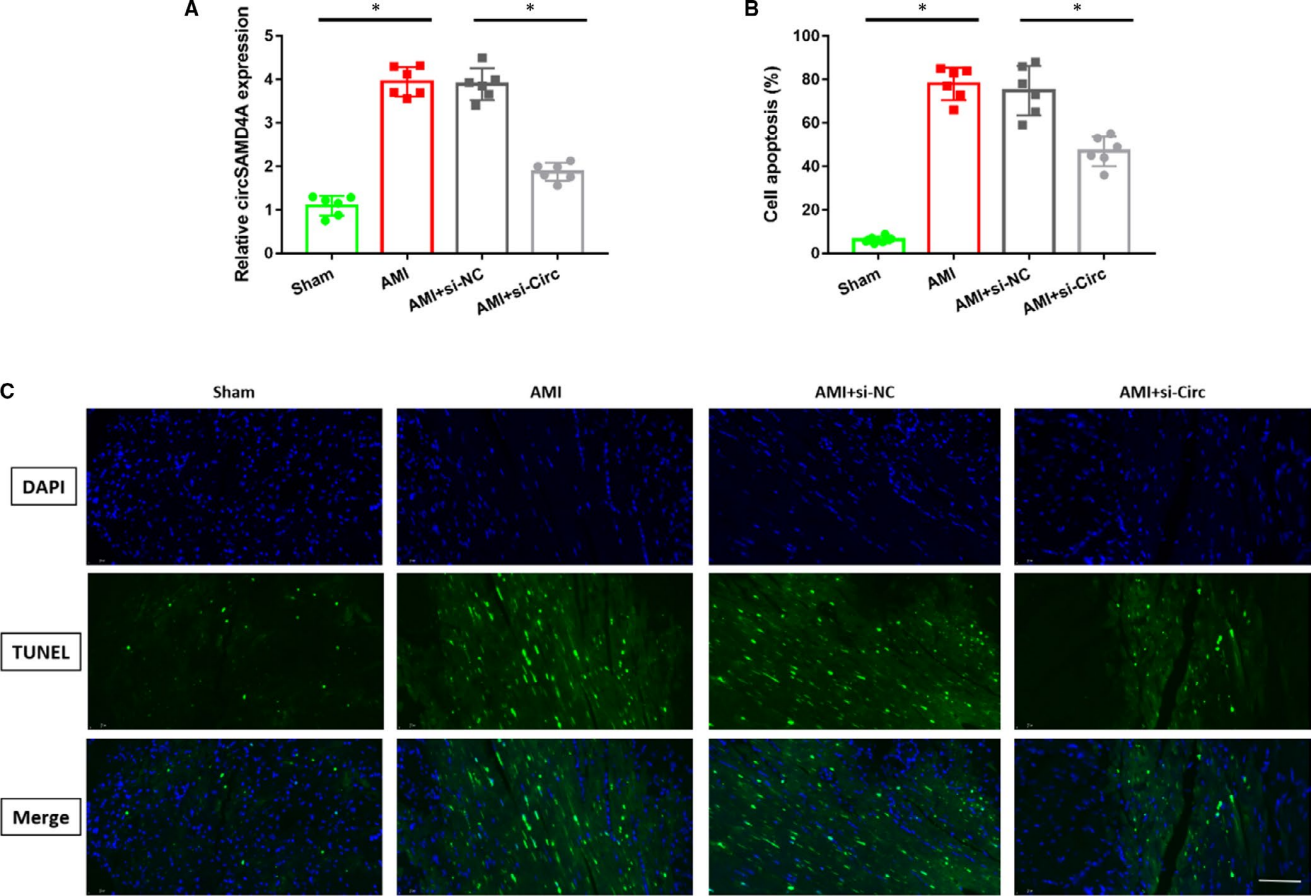
A, Expression of circSAMD4A in sham group (n = 6), AMI group (n = 6) and AMI + si‐Circ group (n = 6). B, The percentage of apoptosis, n = 6. C, Typical TUNEL staining that shows cardiac cell apoptosis, n = 6

To clarify the role of circSAMD4A in AMI, this study firstly established a mouse model of AMI, then separately tested the levels of circSAMD4A in the myocardial cells of the cardiac tissue of the sham group and AMI group. As shown in Figure 1A, the levels of circSAMD4A were significantly higher in AMI group than sham group. There is no evident difference in circSAMD4A expression levels between AMI group and AMI group transfected with si‐NC. However, the circSAMD4A concentration was significantly decreased after transfected si‐CircSAMD4A with AMI group. The results showed that circSAMD4A was overexpressed in AMI.

### 3.2 circSAMD4A can promote apoptosis of cardiomyocytes in vivo

In order to explore the function of circSAMD4A in AMI, we observed the apoptosis of cells by TUNEL staining in this study. Compared with sham group, the numbers of cardiomyocytes apoptosis in AMI group were significantly increased, but inhibiting the circSAMD4A in mice with AMI significantly reduced the numbers of cardiomyocyte apoptosis, as shown in Figure 1B. And it was found that the numbers of TUNEL staining positive cells in AMI group were higher than sham group, and the numbers of TUNEL staining positive cells in AMI + si‐Circ group were lower than AMI + si‐NC group, as shown in Figure 1C. These data indicated that circSAMD4A could regulate cardiomyocytes apoptosis.

### 3.3 Inhibiting circSAMD4A suppresses H/R‐induced cardiomyocyte apoptosis and inflammatory response in H9C2 cells

**Figure 2:**
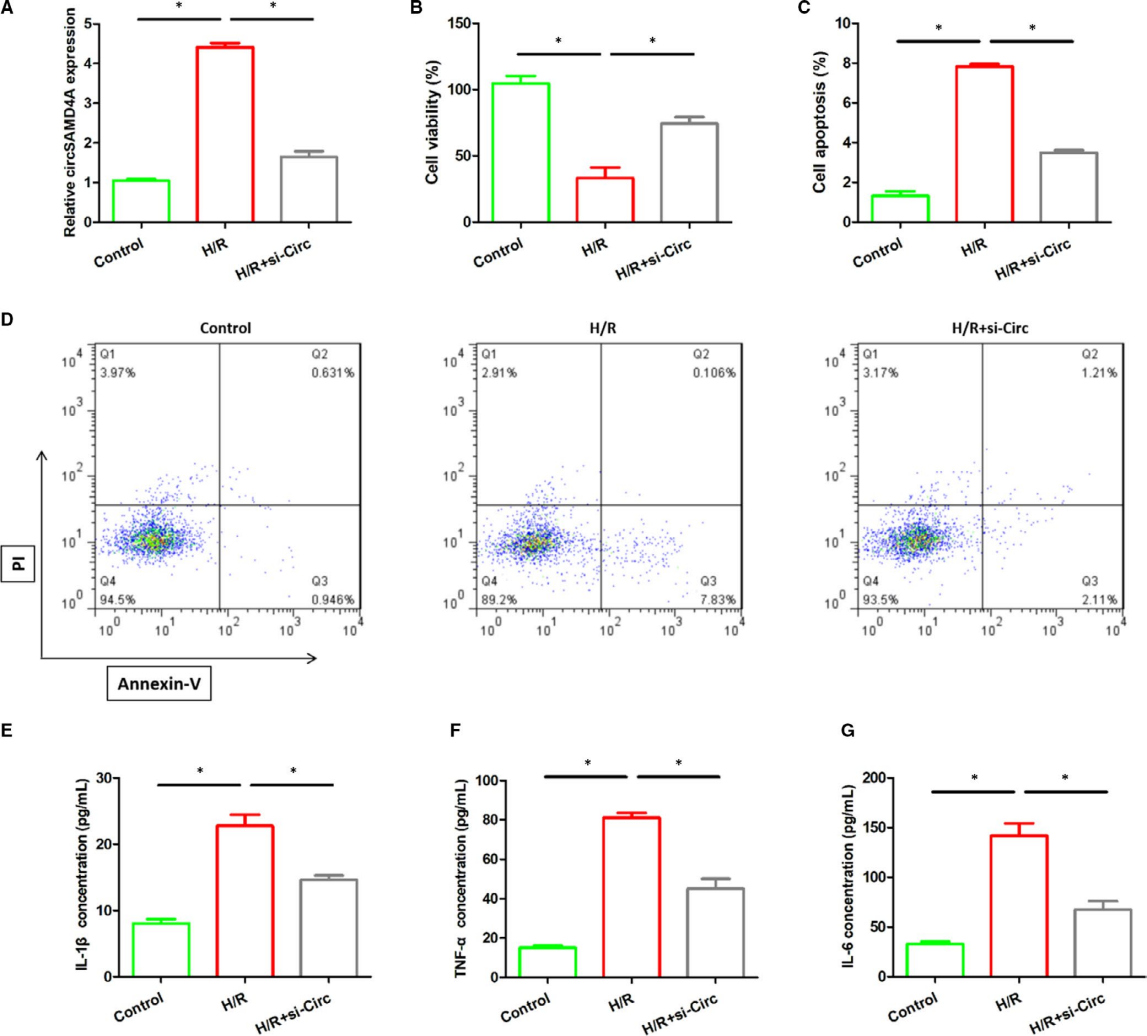
A, The expression of circSAMD4A in the control group, H/R stimulation and H/R + si‐Circ cells. B, MTT assay was used to measure the cell viability after H/R and H/R + si‐Circ treatment. C and D, Annexin V‐PI staining was used to detect apoptosis after H/R and H/R + si‐Circ treatment. E‐G, The expression of IL‐1β, TNF‐α and IL‐6 in the control group, H/R stimulation and H/R + si‐Circ cells

In addition, this study established an AMI cell model and detected the expression of circSAMD4A in cells by real‐time quantitative PCR. As shown in Figure 2A, we observed that the levels of circSAMD4A in H9C2 cells after H/R stimulation were significantly higher than control group. Subsequently, the viability of the cells and the apoptosis of the cells were measured. It showed that the viability of H9C2 cells after H/R stimulation was significantly reduced when compared with the control group, the inhibition of the circSAMD4A made the viability of the cells obviously restored, as shown in Figure 2B. Consistent with the results of the AMI mice, the numbers of apoptosis in H/R‐ induced H9C2 cells were significantly higher than control group, and inhibiting the expression of the circSAMD4A would down‐regulate the numbers of apoptotic cells significantly, as shown in Figure 2C,D.

The role of circSAMD4A in H/R‐induced inflammation was also measured. The results stated that the levels of inflammatory cytokines (IL‐1β, TNF‐α, IL‐6) in H9C2 cells after H/R stimulation evidently become higher than untreated H9C2 cells, but inhibiting the expression of circSAMD4A in H/R‐treated H9C2 cells would dramatically decrease levels of IL‐1β, TNF‐α and IL‐6 (Figure 2E‐G).

The above findings indicated that H/R stimulation might destroy the viability of cardiomyocytes, promote the apoptosis and inflammatory response of cardiomyocytes, but restraining the expression of circSAMD4A can alleviate this damage.

### 3.4 CircSAMD4A targets miR‐138‐5p in H9C2 cells

**Figure 3:**
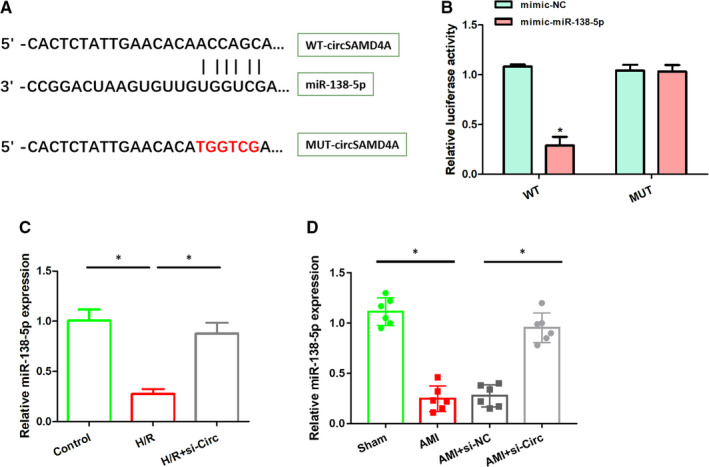
A, Using RNAInter online tool to predict the binding site of circSAMD4A and miR‐138‐5p. B, The relative luciferase activity of WT‐circSAMD4A and MUT‐circSAMD4A after transfection of miR‐138‐5p mimic or NC was detected by dual‐luciferase reporter method. C, The MiR‐138‐5p expression in control group, H/R stimulation and H/R + si‐Circ cells. D, The miR‐138‐5p expression in sham group (n=6), AMI group (n=6), AMI+si‐NC (n=6), AMI+si‐Circ group (n=6).

The bioinformatic method predicts that circSAMD4A binds to miR‐138‐5p. The binding sequence was shown in Figure 3A. And dual‐luciferase report test was used to verify the hypothesis, and it was found that the relative luciferase activity of miR‐138‐5p mimic was significantly reduced in WT‐circSAMD4A compared with the NC group, while there was no significant change in relative luciferase activity in MUT‐circSAMD4A, as shown in Figure 3B. In vitro and vivo experiments, we also noticed that the miR‐138‐5p expression levels were down‐regulated in H/R‐induced myocardial cell injury models, and suppressing the circSAMD4A could up‐regulated the miR‐138‐5p contents, as shown in Figure 3C,D. These results showed that miR‐138‐5p was the target of circSAMD4A.

### 3.5 CircSAMD4A promotes cardiomyocyte apoptosis and inflammatory response by inhibiting the expression of miR‐138‐5p in vitro

**Figure 4:**
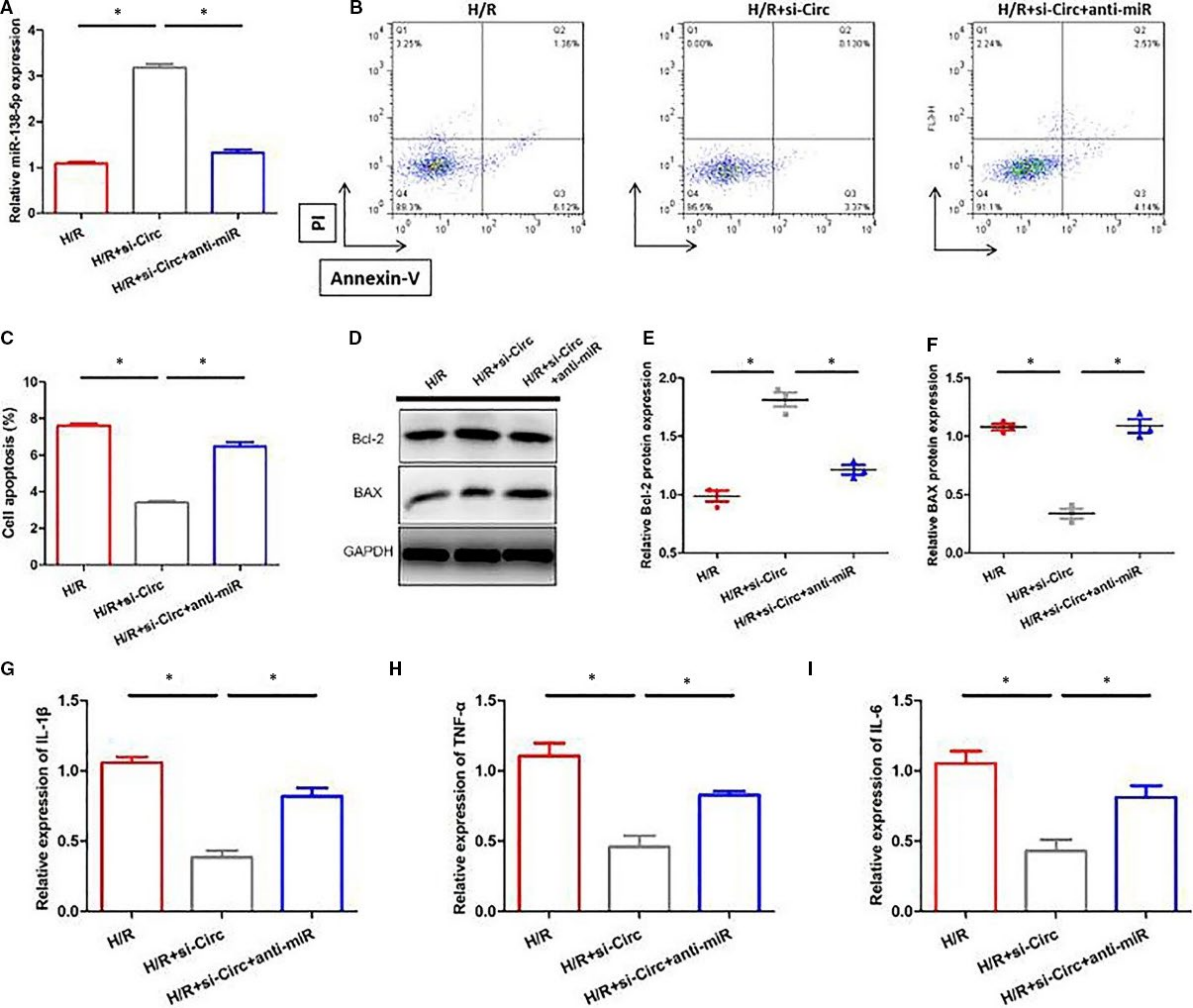
A, Expression of miR‐138‐5p in H9C2 cells after H/R, H/R + si‐Circ and H/R + si‐Circ + anti‐miR treatment. B and C, Annexin V‐PI staining was used to detect apoptosis after H/R, H/R + si‐Cir and H/R + si‐Circ + anti‐miR treatment. D‐F, The detection of apoptosis‐related markers (Bax, Bcl‐2) expression by Western blot and ELISA. G‐I, Expression of IL‐1β, TNF‐α and IL‐6 in H9C2 cells after H/R, H/R + si‐Circ and H/R + si‐Circ + anti‐miR treatment

In order to study the mechanism of circSAMD4A on cardiomyocytes, as shown in Figure 3C, we observed that the levels of miR‐138‐5p in H9C2 cells after H/R stimulation were significantly lower than control group, and the expression of miR‐138‐5p was significantly up‐regulated in H9C2 cells that were inhibited circSAMD4A activity. At the same time, the numbers of cardiomyocyte apoptosis in H9C2 cells that circSAMD4A were knocked out and were stimulated by H/R were lower than in H9C2 cells after H/R treatment, but the levels of miR‐138‐5p were significantly reduced and the numbers of cardiomyocyte apoptosis increased significantly after adding miR‐138‐5p inhibitor to cell system, as shown in Figure 4A‐C. Moreover, the results of Western blot and RT‐PCR showed that inhibiting the expression of circSAMD4A can inhibit the expression of pro‐apoptotic gene Bax and increase the expression of anti‐apoptotic gene Bcl‐2, and the inhibition of circSAMD4A and miR‐138‐5p together would promote the expression of the pro‐apoptotic gene Bax and reduce the expression of the anti‐apoptotic gene Bcl‐2 (Figure 4D‐F). The contents of inflammatory cytokines were further analysed, and the results indicated that the concentrations of IL‐1β, TNF‐α and IL‐6 in H9C2 cells after H/R + si‐Circ treatment were significantly decreased compared with H9C2 cells after H/R treatment, and the concentration of IL‐1β, TNF‐α, IL‐6 in H9C2 cells after H/R + si‐Circ + anti‐miR treatment was significantly increased than H9C2 cells after H/R treatment.

Taken together, circSAMD4A could promote H/R‐induced apoptosis and inflammatory response by directly targeting miR‐138‐5p.

### 3.6 CircSAMD4A suppresses miR‐138‐5p to induce cardiomyocyte apoptosis in vivo

**Figure 5:**
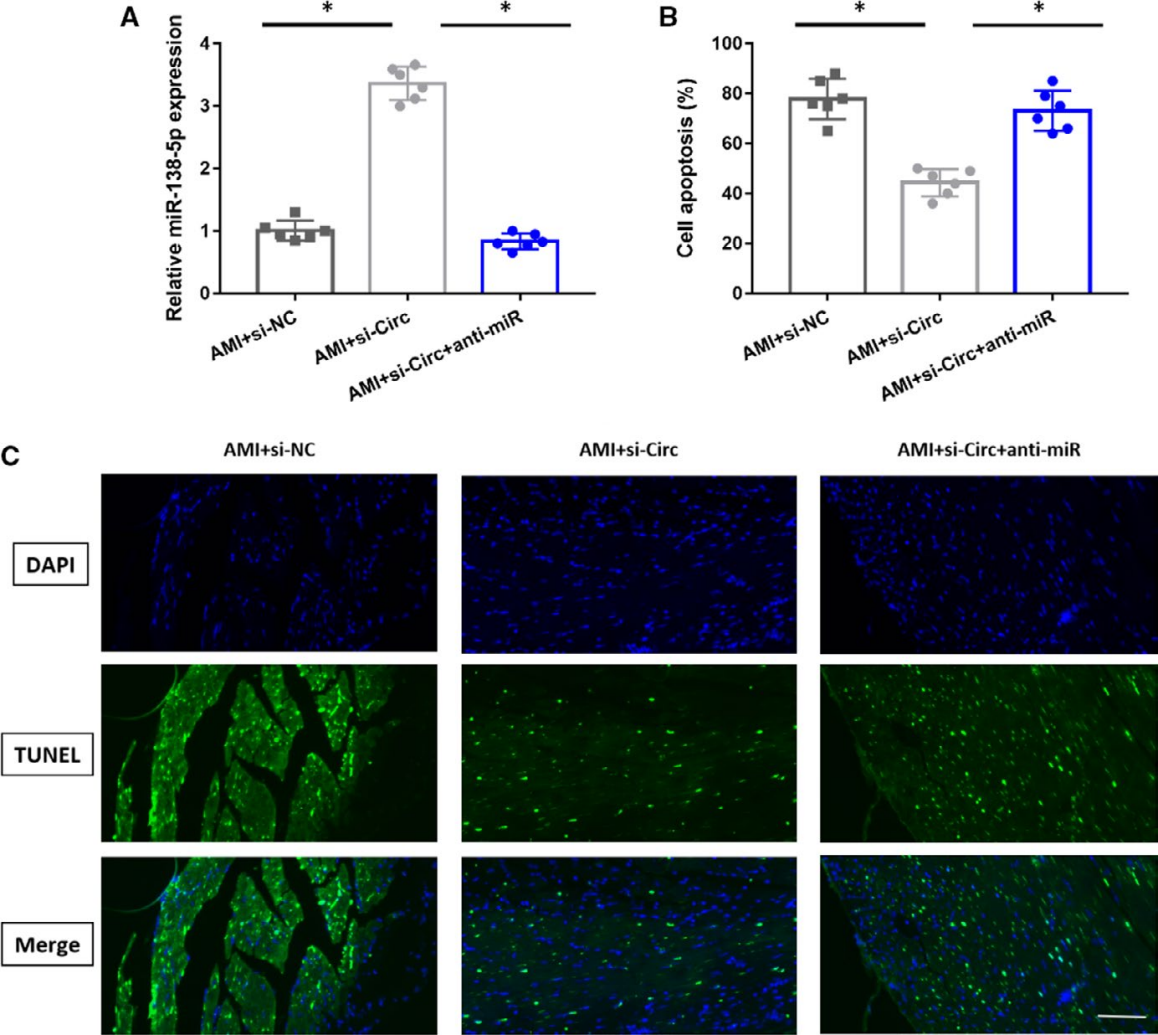
A, The miR‐138‐5p expression in AMI + si‐NC (n = 6), AMI + si‐Circ group (n = 6) and AMI + si‐Circ + anti‐miR group (n = 6). B, The cell apoptosis in AMI + si‐NC (n = 6), AMI + si‐Circ group (n = 6) and AMI + si‐Circ + anti‐miR group (n = 6). C, Typical TUNEL staining that shows cardiac cell apoptosis in AMI mice (bar = 50 µm)

Else, we conducted the vivo experiments to validate the interactions between circSAMD4A and miR‐138‐5p. As shown in Figure 5A,B, the relative miR‐138‐5p expression was significantly up‐regulated but the cell apoptosis ratio was clearly decreased after knocking out the circSAMD4A in AMI mice, which further treated with anti‐miR evidently could decrease the concentration of miR‐138‐5p and augment the ability of cell apoptosis. The similar apoptosis results were also shown in Figure 5C by TUNEL staining. The findings showed that circSAMD4A could target miR‐138‐5p to support cardiomyocyte apoptosis in AMI mice.

## 4 DISCUSSION

AMI is a type of disease that causes serious damage to human life and health, and it imposes a huge economic burden on the development of individuals and society.^14^ It is important to elucidate the molecular mechanism in AMI development. In this study, we tested the level of circSAMD4A in AMI mice and H/R‐stimulated H9C2 cells. First, heart tissue cardiomyocytes in AMI mice and H/R‐induced H9C2 cells performed increased apoptosis and increased levels of circSAMD4A. Second, the circSAMD4A can sponge miR‐138‐5p, thereby enhancing H/R‐induced cardiomyocytes damage by interfering with the apoptosis pathway and inflammatory response by adjusting the expression of inflammatory cytokines.

In recent years, studies have shown that circSAMD4A can target microRNA to regulate biological processes. Liu et al^15^ found that circSAMD4A could control the occurrence of obesity through the miR‐138‐5p/EZH2 axis. In our study, aberrantly expressed circSAMD4A boosted cell apoptosis and inflammation in H/R‐induced H9C2 cells, indicating that circSAMD4A may be involved in H/R‐induced cell damage. However, inhibiting the expression of circSAMD4A would reduce cardiomyocyte apoptosis and inflammatory response in H9C2 cells. In order to understand the mechanism of circSAMD4A regulates cardiomyocyte apoptosis and inflammation development, we conducted in‐depth explorations.

A large number of studies have shown that microRNAs play important role in the progress of H/R‐induced damage, and miR‐138‐5p is included in these microRNAs.^6^ Wang et al^16^ found that miR‐138‐5p was abnormal in myocardial infarction patients and mice, and miR‐138‐5p promoted H/R‐induced cardiotoxicity and regulated cardiomyocyte apoptosis through the SIRT1‐PGC‐1α pathway, which was similar with the role of miR‐138‐5p was involved in H/R‐induced cardiomyocyte apoptosis in this study. IL‐1β, TNF‐α and IL‐6 are linked with the inflammatory reaction of cardiomyocytes in AMI.^17^ Previous studies reported that the levels of IL‐1β were closely related with the impaired myocardial function, and IL‐1β might promote adverse cardiac remodelling during the acute phases of myocardial function and inhibit systolic/diastolic function during the chronic phases of myocardial function.^18^, ^19^ The levels of TNF‐α increased in the local infarct myocardium, which contributed to AMI and cause myocardial cell apoptosis.^8^, ^20^ IL‐6 contributed to the development of infarct size during the early stage of AMI ischaemia‐reperfusion injury.^21^, ^22^ In the present study, we found that the concentration of IL‐1β, TNF‐α and IL‐6 was significantly different between control group and H/R‐induced H9C2 cells, and inhibiting the expression of circSAMD4A in H/R‐stimulated H9C2 cells would ameliorate changed levels of IL‐1β, TNF‐α and IL‐6. After further research, it was found that the circSAMD4A could sponge miR‐138‐5p and regulate the expression of miR‐138‐5p in vivo and vitro. The existing results showed that the simultaneous suppression of the expression of the circSAMD4A and miR‐138‐5p promoted the expression of the pro‐apoptotic gene Bax, reduced the expression of the anti‐apoptotic gene Bcl‐2 and increased the expression of IL‐1β, TNF‐α and IL‐6 compared with the suppression of the circSAMD4A in H9C2 cells. In vivo experiments, we found that inhibiting the expression of circSAMD4A in AMI mice could debase the cell apoptosis, and pre‐treating AMI mice with anti‐miR‐138‐5p could aggrandize the cell apoptosis. We reported the connection of circSAMD4A and miR‐138‐5p in H/R‐induced H9C2 cells and AMI mice for the first time. In the successive research, we will validate the role of circSAMD4A on the cardiomyocyte apoptosis by utilizing more experiment models and explore the effect of exposure time of H/R in vitro on the cardiomyocytes.

Overall, we demonstrated that the circSAMD4A can regulate the expression of miR‐138‐5p to participate in H/R‐induced cardiomyocyte apoptosis and inflammatory response. CircSAMD4A may be a new direction to explore the treatment of patients with AMI.

## CONFLICT OF INTEREST

The authors confirm that there are no conflicts of interest.

## AUTHOR CONTRIBUTION

**Xiaorong Hu:** Investigation (equal); Writing‐original draft (equal). **Ruisong Ma:** Investigation (equal). **Jianlei Cao:** Investigation (equal). **Xianjin Du:** Investigation (equal). **Xinyong Cai:** Project administration (equal); Writing‐original draft (equal); Writing‐review & editing (equal). **Yongzhen Fan:** Project administration (equal); Writing‐original draft (equal); Writing‐review & editing (equal).

## Data Availability

I confirm that I have included a citation for available data in my References section.

## References

[1] Ong SB , Hernández‐Reséndiz S , Crespo‐Avilan GE , et al. Inflammation following acute myocardial infarction: multiple players, dynamic roles, and novel therapeutic opportunities. Pharmacol Ther. 2018;186:73‐87. 29330085 10.1016/j.pharmthera.2018.01.001 PMC5981007

[2] Guo Y , Luo F , Liu Q , Xu D . Regulatory non‐coding RNAs in acute myocardial infarction. J Cell Mol Med. 2016;21:1013‐1023. 27878945 10.1111/jcmm.13032 PMC5387171

[3] Zhou Q , Zhang Z , Bei Y , Li G , Wang T . Circular RNAs as novel biomarkers for cardiovascular diseases. Adv Exp Med Biol. 2018;2018(1087):159‐170. 10.1007/978-981-13-1426-1_13 30259365

[4] Orogo AM , Gustafsson ÅB . Cell death in the myocardium: my heart won’t go on. IUBMB Life. 2013;2013(65):651‐656. 10.1002/iub.1180 PMC4074399 23824949

[5] Frangogiannis NG . Regulation of the inflammatory response in cardiac repair. Circ Res. 2012;110:159‐173. 22223212 10.1161/CIRCRESAHA.111.243162 PMC3690135

[6] Duan J , Yang YU , Liu H , et al. Osthole ameliorates acute myocardial infarction in rats by decreasing the expression of inflammatory‐related cytokines, diminishing MMP‐2 expression and activating p‐ERK. Int J Mol Med. 2015;37:207. 26549213 10.3892/ijmm.2015.2402

[7] Wollenweber T , Roentgen P , Schäfer A , et al. Characterizing the inflammatory tissue response to acute myocardial infarction by clinical multimodality noninvasive imaging. Circ Cardiovasc Imaging. 2014;7:811‐818. 25049056 10.1161/CIRCIMAGING.114.001689

[8] Sun M , Dawood F , Wen WH , et al. Excessive tumor necrosis factor activation after infarction contributes to susceptibility of myocardial rupture and left ventricular dysfunction. Circulation. 2004;110:3221‐3228. 15533863 10.1161/01.CIR.0000147233.10318.23

[9] Han B , Chao J , Yao H . Circular RNA and its mechanisms in disease: from the bench to the clinic. Pharmacol Ther. 2018;187:31‐44. 29406246 10.1016/j.pharmthera.2018.01.010

[10] Zhang W , Li Y , Wang P . Long non‐coding RNA‐ROR aggravates myocardial ischemia/reperfusion injury. Braz J Med Biol Res. 2018;51:e6555. 29694511 10.1590/1414-431X20186555 PMC5937723

[11] Hu YH , Sun J , Zhang J , Hua FZ , Liu Q , Liang YP . Long non‐coding RNA ROR sponges miR‐138 to aggravate hypoxia/reoxygenation‐induced cardiomyocytes apoptosis via upregulating Mst1. Exp Mol Pathol. 2020;114:104430. 32240614 10.1016/j.yexmp.2020.104430

[12] Geng HH , Li R , Su YM , et al. The circular RNA Cdr1as promotes myocardial infarction by mediating the regulation of miR‐7a on its target genes expression. PLoS One. 2016;11:e0151753. 26998750 10.1371/journal.pone.0151753 PMC4801407

[13] Zhao B , Li G , Peng J , et al. CircMACF1 attenuates acute myocardial infarction through miR‐500b‐5p‐EMP1 axis. J Cardiovasc Transl Res. 2020;19. 10.1007/s12265-020-09976-5 32162171

[14] Steg PG , James SK , Atar D , et al. ESC guidelines for the management of acute myocardial infarction in patients presenting with ST‐segment elevation: the task force on the management of ST‐segment elevation acute myocardial infarction of the European Society of Cardiology (ESC). Eur Heart J. 2012;2012(33):2569‐2619. 10.1093/eurheartj/ehs215 22922416

[15] Liu Y , Liu H , Li Y , Mao R , Yang H , Zhang Y , Zhang Y , Guo P , Zhan D , Zhang T . Circular RNA SAMD4A controls adipogenesis in obesity through the miR‐138‐5p/EZH2 axis. Theranostics. 2020;10(10):4705–4719. 32292524 10.7150/thno.42417 PMC7150479

[16] Wang C , Sun X , Qiu Z , Chen A . MiR‐138‐5p exacerbates hypoxia/reperfusion‐induced heart injury through the inactivation of SIRT1‐PGC‐1α. Inflamm Res. 2019;68:867‐876. 31312857 10.1007/s00011-019-01268-2

[17] Nian M , Lee P , Khaper N , Liu P . Inflammatory cytokines and postmyocardial infarction remodeling. Circ Res. 2004;94:1543‐1553. 15217919 10.1161/01.RES.0000130526.20854.fa

[18] Chen B , Geng J , Gao SX , et al. Eplerenone modulates interleukin‐33/sST2 signaling and IL‐1β in left ventricular systolic dysfunction after acute myocardial infarction. J Interferon Cytokine Res. 2018;38(3):137. 29565745 10.1089/jir.2017.0067

[19] Toldo S , Mezzaroma E , Bressi E , et al. Interleukin‐ 1beta blockade improves left ventricular systolic/diastolic function and restores contractility reserve in severe ischemic cardiomyopathy in the mouse. J Cardiovasc Pharmacol. 2014;64:1‐6. 25006675 10.1097/FJC.0000000000000106

[20] Yuan M , Zhang L , You F , et al. MiR‐145‐5p regulates hypoxia‐induced inflammatory response and apoptosis in cardiomyocytes by targeting CD40. Mol Cell Biochem. 2017;431:123‐131. 28281187 10.1007/s11010-017-2982-4

[21] Jong WMC , Ten Cate H , Linnenbank AC , et al. Reduced acute myocardial ischemia–reperfusion injury in IL‐6‐deficient mice employing a closed‐chest model. Inflamm Res. 2016;65:489‐499. 26935770 10.1007/s00011-016-0931-4 PMC4841857

[22] Empana JP , Jouven X , Canoui‐Poitrine F , et al. C‐reactive protein, interleukin 6, fibrinogen and risk of sudden death in European middle‐aged men: the PRIME study. Arterioscler Thromb Vasc Biol. 2010;30:2047‐2052. 20651278 10.1161/ATVBAHA.110.208785

